# Structural and Material Determinants Influencing the Behavior of Porous Ti and Its Alloys Made by Additive Manufacturing Techniques for Biomedical Applications

**DOI:** 10.3390/ma14040712

**Published:** 2021-02-03

**Authors:** Magda Dziaduszewska, Andrzej Zieliński

**Affiliations:** Biomaterials Technology Division, Institute of Machines Technology and Materials, Faculty of Mechanical Engineering and Ship Building, Gdańsk University of Technology, 80-233 Gdańsk, Poland; andrzej.zielinski@pg.edu.pl

**Keywords:** additive manufacturing, selective laser melting, titanium, titanium alloys, scaffolds, mechanical and biological properties, bioactivity, antibacterial efficiency, mechanical strength, corrosion resistance

## Abstract

One of the biggest challenges in tissue engineering is the manufacturing of porous structures that are customized in size and shape and that mimic natural bone structure. Additive manufacturing is known as a sufficient method to produce 3D porous structures used as bone substitutes in large segmental bone defects. The literature indicates that the mechanical and biological properties of scaffolds highly depend on geometrical features of structure (pore size, pore shape, porosity), surface morphology, and chemistry. The objective of this review is to present the latest advances and trends in the development of titanium scaffolds concerning the relationships between applied materials, manufacturing methods, and interior architecture determined by porosity, pore shape, and size, and the mechanical, biological, chemical, and physical properties. Such a review is assumed to show the real achievements and, on the other side, shortages in so far research.

## 1. Introduction

Scaffolds, in terms of a three-dimensional porous structure that provides support and allows cells to adhere, proliferate, and differentiate, are used as bone substitutes for large segmental bone defects to restore the functionality of bone [1]. Scaffolds should meet certain material, structural, and mechanical criteria to provide cell interaction and structural support [2]. Mechanical properties similar to those of the host tissue [3,4], 3D architecture with interconnected pores designed in size and shape [2,5], as well as surface properties providing cell adhesion, proliferation, and differentiation, define the quality of the effective bone scaffold [6]. Apart from the biocompatibility, noncytotoxicity, and biodegradability at a rate matching the rate of the remodeling process, the bone scaffold should mimic bone morphology, structure, and function to optimize implant–tissue integration [7]. The characteristic of a scaffold suitable for bone regeneration is based on the phase composition, crystalline structure, and microstructure determined by chemical composition, fabrication, heat, plastic, and surface treatment [8]. So far, research shows that the mechanical and biological properties highly depend on geometrical features of structure (pore size, pore shape, porosity), surface morphology, and chemistry of scaffolds [6].

One of the biggest challenges in tissue engineering is the manufacturing of porous structures that are customized in size and shape and that mimic natural bone structure [9]. Scaffolds dedicated for load-bearing implants seem to be a challenge, since the material and architecture of implants should maintain an appropriate relationship between strength and biological features.

The titanium alloys are characterized by excellent biocompatibility, corrosion resistance, high strength-to-weight ratio, and good fatigue resistance. However, the significant mismatch of moduli between titanium and bones may contribute to stress shielding, leading to implant loosening [10]. According to Wolff’s law, the remodeling process of the bone is strictly correlated with its responses to the load under which it is placed, and an implant with higher elasticity modulus starts bearing most of the load, which leads to a reduction in bone density and its resorption. As a consequence, further revision surgery may be required [11]. Therefore, the idea is to manufacture porous titanium structures with a controllable Young’s modulus, and open spaces promoting bone tissue ingrowth is a perspective idea [12].

Different methods have been proposed to develop a titanium porous structure. Conventional manufacturing approaches, mostly based on powder metallurgy, such as metal injection molding or the space-holder method, are subtractive methods where parts of the material are removed from an initial block to achieve the desired shape. Although those fabrication technologies have shown great promise in fabricating scaffolds, certain limitations, such as the impossibility to manufacture highly complex parts with controllable geometry (pore shape, interconnectivity), precious dimensions (pore and strut size), and predictable mechanical properties, remain. Additionally, due to the extensive production line involving tooling and manufacturing sequences, the traditional methods become cost- and time-consuming [8,13,14].

The most promising rapid prototyping techniques, introduced to overcome conventional manufacturing limitations, are called additive manufacturing (AM) processes that deliver the final three-dimensional object via deposition of overlying layers. An important improvement is the possibility to create custom-made products using computer-assisted technologies (CAD), starting from the patient’s body medical images. AM methods allow production of complex geometry with internal and external macro- and microarchitecture, dimensional accuracies, significantly lower defect rates, and enhanced mechanical properties, all within a shorter time and with lower materials wastes [13].

According to Maroulakos [5], AM technologies can be divided into three main categories based on material deposition methods: (i) laser-based machining, (ii) direct printing technologies, and (iii) nozzle-based systems. Yuan et al. [15] classified AM technologies based on the material resources as follows: (i) using metal powders, (ii) using wires, (iii) using sheets. The American Society for Testing and Materials (ASTM) provides a classification of all AM technologies (“ASTM F42-Additive Manufacturing”) into seven categories: (i) binder jetting (BJ), (ii) direct energy deposition (DED), (iii) material extrusion (ME), (iv) material jetting (MJ), (v) powder bed fusion (PBF), (vi) sheet lamination, and (vii) vat photopolymerization [16]. In this paper, the authors are focused on the most common metal-based additive manufacturing processes employed for the manufacturing of scaffolds, especially the selective laser melting (SLM), selective laser sintering (SLS), electron beam melting (EBM), laser engineered net shaping (LENS), fused deposition modeling (FDM), metal injection molding (MIM), direct ink writing (DIW), and 3D fiber deposition (3DF).

The issue of parts and scaffolds made through additive manufacturing (AM) has been often characterized in the literature. Some reviews collected and analyzed data about different metallic and nonmetallic materials, and various AM techniques [17,18,19,20,21], considering especially the formation of the microstructure, mechanical properties, and process parameters. Several reviews described AM of only scaffolds. In [22], the topological design and manufacturing processes of various types of porous metals, in particular titanium alloys, biodegradable metals, and shape memory alloys, were presented. Similar subjects were reviewed in [23] with a special emphasis on the open-cellular structural design for porous metallic implant applications, mechanical properties towards fatigue-tolerant design and fabrication achieved by AM technology. Only the metallurgical aspects, allowing prediction of microstructures and mechanical properties of laser and electron beam additively manufactured porous metallic implants, were shown in [11]. In a similar review [24], the effects of material type, processing, geometrical design, and post-AM treatments on the mechanical properties, biodegradation behavior, in vitro biocompatibility, and in vivo bone regeneration performance of different additively manufactured porous metals were presented. Finally, there were some papers especially devoted to Ti and its alloys. In [25], analyses of AM process parameters and microstructural and mechanical properties of Ti-6Al-4V alloy were presented. In another review [26], recent progress on fatigue behavior of additively manufactured Ti-6Al-4V alloy was shown. Liu et al. [27] focused on Ti-6Al-4V alloy fabricated through additive manufacturing, directed energy deposition, selective laser melting, and electron beam melting. Singh et al. [28] focused on the optimization of process parameters of SLM-made Ti alloys and Ti-based composites. Li et al. [29] described various factors that could influence biological behavior such as pore size, porosity, pore structure, and surface modification for additively manufactured scaffolds. In the last review cited here [30], the powder metallurgy with the use of a space holder for Ti implants was shown.

All recent reviews have been either devoted to some specific subjects or process optimization or overall presentation of AM methods. The objective of this review is to present the latest advances and trends in the development of titanium scaffolds concerning the relationships between applied materials, manufacturing methods, and interior architecture determined by porosity, pore shape, and size and the mechanical, biological, chemical, and physical properties. Such a review is assumed to show the real achievements and, on the other side, shortages in research so far. Even more importantly, it fills to some extent a gap in this specific science field, showing not only the main requirements of the titanium implants, different fabrication AM technologies, and titanium and its alloys proposed for scaffolds, but also a relatively new approach in design and manufacturing of porous Ti and its alloys, i.e., relationships between all microstructural factors, regardless of their materials, by which method they are manufactured, and their mechanical, biological, and chemical properties.

The methodology to prepare this review was based on several inclusion factors. The main factors included the subject of the study (Ti or Ti-base porous implants; scaffolds), publication data (the last 10 years, as a rule), addressed readers (materials, mechanical, and biomedical engineers and scientists, and physicians specialized in implantology of load-bearing implants, i.e., dental, joint and maxillofacial implants), peer review (only papers in eminent journals from the JCR list were taken into account), reported outcomes (the relationships between microstructure and mechanical, biological and chemical properties), and searched databases (Elsevier, Springer, MDPI). The originality of this review is in its limitation for all titanium-made scaffolds (not only Ti-6Al-4V), their additive manufacturing approaches, surface treatment, and properties, with an emphasis put on relationships between manufacturing technique, material microstructure, and mechanical, biological, and chemical properties.

## 2. Biological Background—Formation and Regeneration Process of Bone

The present understanding of the biomechanical processes that affect healing is based on the natural bone formation process, which can be divided into two stages: primary and secondary bone healing. Primary bone healing occurs when the fracture is rigidly stabilized and the gap is less than 0.1 mm. The bone formation is provided directly by osteoclasts and osteoblasts. Secondary bone healing occurs when only a small movement at the fracture appears [31]. In general, multievents, such as blood clotting, inflammatory response, fibrocartilage callus formation, intramembranous and endochondral ossification, and bone remodeling are involved in the secondary bone fracture repair [32]. In one study, another type of fracture, called the critical size bone defect, is mentioned; it is defined as the large cavity in a bone that cannot regenerate itself naturally [33]. In such a situation, bone repair may delay bonding or even stop fixation, and substitutes for bone defect treatments can be necessary. A hematoma initiates a proinflammatory cascade [34,35]. Additionally, the environment with low pH and a raised level of lactate leads to increased activity of angiogenic factors [36]. Bone formation starts with the change of the monocyte M1 to anti-inflammatory macrophages (M2), which secrete a battery of cytokines and growth factors to promote tissue repair and angiogenesis [37]. In the fracture gap, the progenitor cells undergo chondrogenesis to form bone [35,36]. Calcification is a process of direct bone formation in which osteochondral progenitors differentiate directly into osteoblasts. Osteoblasts synthesize extracellular matrix containing collagen type I and coordinate matrix mineralization. The conversion of cartilage to the bone during endochondral ossification occurs simultaneously with the appearance of blood vessels. During bone remodeling, the cartilage becomes fully transformed into the trabecular bone and remodeled into the cortical bone, which fills the full defect of the fracture and is almost indistinguishable in form and function from the native bone [38].

**Figure 1:**
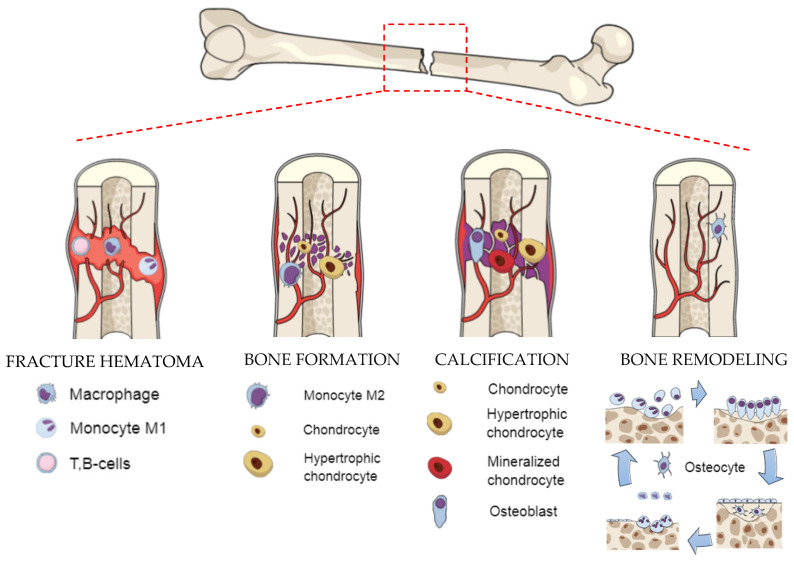
Scheme of natural bone formation [34,35,36,37].

Knowledge about the biological structure of bone and the natural healing process is important in terms of improving therapeutic approaches to tissue engineering. The structure of bone contains bone tissue (macro scale), osteons (100 µm) with Haversian canals, fiber patterns (50 µm), fibers (5 µm), mineralized collagen fibrils (500 nm), collagen, and hydroxyapatite (HAp) [39]. The organic component in the extracellular matrix (collagen type I) gives the bone tensile strength, whereas the inorganic component brings out the compressive strength [39]. The bone is in a dynamic balance related to the modeling and remodeling process [40]. Bone modeling is associated mainly with the growth period (continued in adulthood) and responsible for the skeletal mass increase and skeletal form changes. It increases bone strength and stiffness by improving geometric properties [41]. In contrast, bone remodeling is a lifelong process responsible for maintaining bone function by a continuous replacement of the damaged bone by a new one without mass change [40]. In the bone modeling process, bone resorption and bone formation occur on separate surfaces, whereas bone remodeling is preceded by resorption and contains a coupled process [32,38,42,43,44]. Figure 1 presents the scheme of natural bone formation.

## 3. The Main Requirements for Bone Scaffolds

### 3.1. Biomechanical Properties

Scaffolds for bone regeneration should meet certain material, structural, and mechanical criteria to provide cell interaction and structural support [2]. The mismatch between the mechanical properties of bone and those of the biomaterial could induce an uneven stress distribution, leading to bone resorption around the metal implants, increased risk of fractures in the weakened bone, and relaxation of the implants. This phenomenon is referred to as “stress shielding”, which usually reduces the longevity of implants [45,46]. As the mechanical properties of bone depend on age, activity, and disease status, proper matching constitutes a major challenge for tissue engineering [2]. The most critical mechanical properties of human cortical and trabecular bone are presented in Table 1.

### 3.2. Design

**Figure 2:**
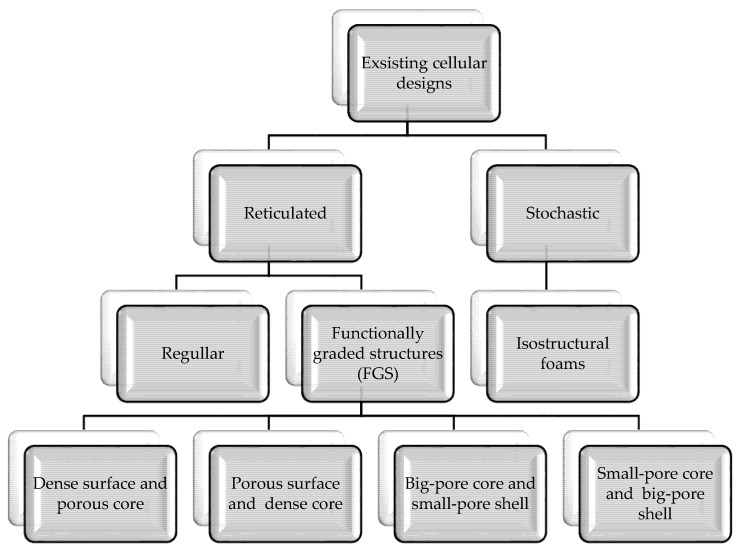
Collection of existing cellular designs.

Porous structures exist in many forms, both stochastic (irregular), such as expanded or reticular (regular) foams, or as grid structures or meshes with continuously repeating elementary cells (Figure 2) [11,52,53].

Stochastic (isostructural) open-cell foams are characterized by randomly shaped and sized pores with irregularly repeated unit cells, stochastically connected and orientated struts. The structure of stochastic foams is often heterogeneous, which means that foams are strong in some regions but weak in others [51,54]. Weak regions greatly reduce mechanical efficiency. The improvement of mechanical properties can be achieved through the structures with unit cells in an ordered hierarchy [53,55]. Reticulated structures consist of repeating elementary cells leading to a regular and periodic form. The pores have a uniform size and shape, while the struts are connected and oriented according to certain rules in regular or gradient porosity [55]. The specific unit configurations can be designed through a CAD program or selected from the archive with main unit structures [53,56]. Regular porosity means symmetrical pore structure characterized by one shaped and one sized pore. For comparison, scaffolds with gradient porosity have an asymmetrical pore structure and allow one to obtain a structure similar to the macrostructure of bone with appropriate mechanical properties [57]. As means to achieve the balance between mechanical and mass transport properties, scaffolds with hierarchical porous structures have gained much attention. One can distinguish two main types of functionally graded porosity: fully porous (small-pore core and big-pore shell/big-pore core and small-pore shell) or partly porous (dense surface and porous core/sense core and porous surface) [6,57]. Large pore sizes in the outer region and smaller pore sizes in the inner region support osteoblasts’ adhesion and enhance the transport of nutrients and oxygen [53]. The selection of the appropriate form depends on the expected functionality.

### 3.3. Porosity

Porosity is defined as the percentage of overall voids’ volume in solid material, and its value depends on pore and struts size as well as porous structures [39]. Porosity supports cell migration, determines the transport of oxygen and nutrients, and provides surface areas for new tissue ingrowth [58]. In general, an increase in pore size over the height of the unit cell corresponds to an increase in scaffold porosity [59]. The idea is to design constructions using as little material as possible, simultaneously preserving biomimetic character (50–90% porosity) and strength requirements, since compressive properties of all the porous structures increase with structure relative density [60]. Typically, high porosity scaffolds (>70% porosity) have been shown to possess better bone ingrowth than low porosity scaffolds (<70% porosity) [39]. In most cases, it has been observed that as the percentage porosity increases, the cellular response also increases. For example, in in vivo studies [61,62], a positive effect of increased porosity on osteoconductive properties was observed. Moreover, bigger pores in the shell part of the scaffold supported better nutrient diffusion within smaller pores [63]. Furthermore, tissue development inside the scaffold is also determined by the microporosity of the scaffold [64]. To increase surface area, support the adhesion of the osteoblast, promote vascularization, and improve osteointegration, the architecture should ensure proper interconnectivity between the pores [57]. Open porosity is also important for the diffusion of essential nutrients, oxygen, and extracellular fluid in and out of the cellular matrix. The factors that favor cell ingrowth (such as high porosity or larger pore sizes on outer surfaces) conflict with the need for strong mechanical properties. High porosity decreases the Young’s modulus and provides better matching of mechanical properties to the values of native bone. However, high porosity also decreases mechanical strength and stability, especially in scaffolds dedicated to load-bearing orthopedic applications [65]. Due to the fact that the properties of natural bones vary greatly and depend on many factors, i.e., place in the body, age, sex, and health, an optimal relation between mechanical and biological performance is difficult to achieve. To maximize the mechanobiological response of a porous material, the computer simulation with patient-specific finite element models of bones should be evaluated.

### 3.4. Pore and Strut Size

Research has shown that the minimal pore size for promoting bone ingrowth is in the range of 100–150 µm [47,66,67,68]. The minimum value was determined based on the size of osteoclasts (100 μm), which are responsible for bone resorption processes [67]. In the case of smaller pore size, single cells may extend over and bridge the pores stopping cells from ingrowing, as well as limit diffusion of wastes and nutrient supply to the cellular network. Pore sizes of 200–400 μm were thought to increase osteoblast attachment, migration, and proliferation [51]. Pores larger than 300 μm are believed to favor vascularization capillaries’ formation. Better vascularization promoted the osseointegration process [39,47,69]. The authors also reported also that porosity with pore size exceeding 900 μm performed inefficient cell bridging [53]. In turn, scaffolds with smaller pores were considered to have a larger surface area and therefore more space for bone tissue ingrowth [53]. Due to a compromise between bone growth, vascularization, mechanical strength, and permeability, the optimum pore size was supposed to range between 300 and 600 μm [47,53,70]. Considering only scaffolds made by the SLM, in [71], the authors studied the effects of different unit cell types (tetrahedron and octahedron) and pore size (500 μm and 1000 μm) on fatigue properties. As expected, scaffolds with 1000 μm pore resulted in lower compressive properties and shorter fatigue lives compared to those with 500 μm pore. Struts with different diameters and positions may determine a different failure mechanism, and as previously reported, in the unit cells with vertical struts, the breakdown of one strut resulted in the collapse of the whole unit cell [60]. In general, the density and load-bearing capacity of the samples increase with strut diameter, whereas the strength is exponential relative to the apparent density.

### 3.5. Pore Shape

There have been several studies focusing on the influence of unit cell shape [71,72]. As shown, mechanical properties, both static and fatigue, slightly vary for different unit cell shapes [60,73,74]. What is more, the pore structure affects bone ingrowth [39]. Traditionally, some basic unit shapes are based on cube and honeycomblike structures [75]. Furthermore, there are others based on a CAD model with shapes related to the number of struts and their orientation at different angles [60,76]. The cubic structure is characterized by rectangular pores in a vertical orientation, whereas the pyramidal basic structure exhibits trapezoidal pores in both directions of the *z*-axis. Another basic structure is marked by diagonally oriented struts and checked pores [61]. The strength of the porous structure is obtained, among others, due to the orientation of the struts relative to the load. Yavari et al. [76,77] stated that the diamond, truncated hexagon, and cubic cells demonstrated the highest static and fatigue properties. They showed that the diamond crystal lattice remained stable under directional compressive forces, even with high porosity. While in cells with struts oriented at different angles, there were additional bending forces that caused tensile stresses adversely affecting mechanical properties. Ahmadi et al. [60] also reported that the architecture consisting of supporting struts (such as in a truncated cube) distributed and transmitted forces better. Another study [70] showed that the advantage of a diamond cell shape also based on its crystal lattice consisted only of obtuse angles (109.5°). This is suitable for the SLM process since the sharp angles are often exposed to damage due to adhesion to a nearby strut in the melting phase. Van Bael et al. [72] kept attention to the impact of the number of angles, suggesting that with a larger number of them, such as in the case of hexagonal pores compared to triangular or rectangular, more areas were bridged.

The main requirements of the bone scaffold are shown in Table 2.

## 4. Fabrication Methods of the Titanium Scaffolds and Effects of Manufacturing Errors

### 4.1. Conventional Methods

#### 4.1.1. Powder Metallurgy (PM)

The porosity and pore size are dependent on the kind of space holder and the ratio of the titanium biomaterial to a space holder. As the space holders, NaCl [80,81,82], sugar crystals [83,84,85], polypropylene carbonate [86], magnesium powder [87,88], carbonates [89,90,91,92], carbamide [93,94], and Mo wire [95] were used. The titanium hydride, which could decompose at elevated temperature, was also applied in powder metallurgy. In [96], the Ti scaffold of a porosity 42 vol % and compressive strength of 48 MPa was obtained by sintering with the use of methylcellulose as the binder and TiH_2_ powder as the Ti source. In [97], using TiH_1.924_, the scaffolds possessing pores in the range of 300–600 μm were fabricated. Titanium hydride resulted in higher surface roughness and higher microporosity than in pure titanium. The effect of the space holder fraction was also analyzed. Xu et al. [89] observed, for the Ti-35Zr-28Nb scaffold, that the porosity increased from 50% to 65% when the NH_4_HCO_3_ volume content was increased from 63% to 79%, and the average pore size enlarged from 230 µm to 430 µm. For the titanium scaffolds sintered at 1200 °C for 3 h, the carbamide space holder showed a maximum specific surface area at its addition in 60–65 vol % [93]. An increase in sugar pellets’ amount from 30% to 70% brought out porosity from 21% to 55% [84,85]. For Mg space holders, the porosity of 30–50% exactly corresponded to the volume amount of Mg [88]. The obtained results show that the volume content of the space holder is only roughly close to the porosity and for each alloy and a pore initiator, the porosity can be particularly designed. The presence of a space holder has been postulated to affect the pores’ size. In [80], for porous titanium porosity ranged between 58% and 77%, with dual size range, large pores from 500 to 1000 μm resulted from the NaCl particles, and smaller pores of 1–10 μm resulted from the powder sintering. The obtained porosity of 70 vol % was associated [81] with a structure consisting also of the micro- (<10 μm) and macrointerconnected pores (300–400 μm). The size of the space holder sometimes determined the pore size. In [83], the 1:1 Ti/sugar ratio led to the porosity of about 72% with a pore diameter of 0.8–1.0 mm equaling the diameter of sugar crystals. The powder metallurgy without space holders was seldom attempted [91,98,99].

In [99], porous scaffolds were fabricated as agglomerates of Ti and Ti–10Nb–3Mo alloy particles with a solid core and porous surface layers. The gradient scaffolds, consisting of inner, middle, and outer layers, were also obtained by Fan et al. [100]. The liquid foaming method [101], based on putting the Ti slurry with a binder and antifoaming agent in the mold, drying and sintering at 1300 °C, resulted in a novel porous titanium scaffold with a three-dimensionally hierarchical porous structure of porosity 76% and macropores with pore size larger than 100 µm, micropores with a size of about 10 µm, and networklike nanopores. The scaffold compressive strength and Young’s modulus of the porous Ti scaffold were 23.6 MPa and 2.1 GPa, respectively.

#### 4.1.2. Freeze Casting

In [102,103], the titanium scaffolds with centrosymmetric pore channels in the radial direction were fabricated by freeze-casting; the use of TiH_2_ contents ranged between 20% and 30%. The porosity achieved was 39–53%, pore size 54–113 µm, Young’s modulus 1.4–4.1 GPa, and compressive strength 250–450 MPa. In other research [104], titanium scaffolds with the long-range lamellar structure were obtained using a novel bidirectional freeze-casting method. The porosity and pore size ranged from 67% to 50% and 80 μm to 67 μm, respectively. The compressive strength and stiffness increased from 58 MPa to 162 MPa and from 2.5 GPa to 6.5 GPa, respectively. The multiscale porosity was obtained for the Ti-6Al-4V scaffolds by combining dynamic freeze-casting with microarc oxidation (MAO) [105]. The size of pores ranged from 71% to 51%, pore sizes were 426 to 311 µm, and the yield compressive strength and elastic modulus of porous Ti6Al4V scaffolds increased from 76 to 223 MPa and from 3.8 to 17.8 GPa, respectively. In [106], for the porous titanium scaffolds prepared by freeze-casting, the sintering temperature significantly influenced the porosity and the mechanical properties of the titanium scaffolds. The porosity decreased from 6% to 20% as the sintering temperatures increased from 800 to 1100 °C. The scaffolds had pore sizes ranged from 2 to 20 µm. The elastic modulus was between 2 and 7 GPa, and the compression strength of the scaffolds exceeded 1000 MPa as the sintering temperature was above 1000 °C. The increase in titanium strength could be mainly attributed to a decrease in porosity.

#### 4.1.3. Polymeric Sponge Replication

In [107], the porous Ti scaffolds had uniform porous structure and completely interconnected macropores about 365 μm in size, and two different sizes of micropores, 45 and 6 μm, were also found in the skeleton of the scaffold. Compressive strength of 84 MPa was achieved for a porosity of 66%. In [108], a new type of porous Ti-based alloy scaffold with a porosity of about 75% and interconnected pores in the range of 300–1000 μm was fabricated with Ti-Nb-Zr powders. This porous scaffold exhibited a compressive strength of 14.9 MPa and an elastic modulus of 0.21 GPa, resembling the mechanical properties of natural human cancellous bone obtained in this study, which could be potentially used for bone tissue engineering application. In another research study [109], two kinds of porous titanium scaffolds with different porosities (75% and 88%) and pore sizes (360 μm and 750 μm) were manufactured. Both of the scaffolds exhibited good compressive strength (24.5 MPa and 13.5 MPa) and a low elastic modulus (0.23 GPa and 0.11 GPa). Porous specimens were prepared from a slurry containing 45 vol % TiH_2_ powder. Macropores were sized in the range of 100–600 µm and had rounded shapes, appropriate for the ingrowths of the new-bone tissues and the transport of the body fluids. The compression strength was 24 MPa for 75% porosity [110].

### 4.2. Additive Manufacturing Methods (AM)

#### 4.2.1. Selective Laser Melting (SLM)

An additive manufacturing, such as the SLM technique, becomes a promising opportunity for obtaining structures with controlled architecture, with porosity, pore shape, and size improving implant stability and implant–cell interaction [53,111]. The obtained implants sensibly depend on process parameters and, on the other hand, on the alloy composition and structure. All of these determine the heat flow, the energy necessary for melting and recrystallization, heating and cooling gradients, etc. The great advantage of laser-assisted methods is the possibility to design and manufacture complex structures. In [45], scaffolds with 66–79% of porosity produced by SLM showed biomimetic structure design and customized mechanical properties. The continuous functionally graded porous titanium scaffolds could be also manufactured by the SLM based on the Schwartz diamond unit cell and the strut size of 483–905 μm [112]. The multilayered fully porous scaffold mimicking the morphology of the bone was obtained using SLM [9]. The designed structure also affects the porosity. The SLM resulting in scaffolds of new β-Ti-35Zr-28Nb alloy [113] showed the porosity values of 83% for the FCCZ structure (face-centered cubic unit cell with longitudinal struts) and 50% for the FBCCZ structure (face- and body-centered cubic unit cell with longitudinal struts). In [114], the trabecularlike porous Ti-6Al-4V scaffolds with varying irregularities (0.05–0.5 μm) and porosities 49–74% designed through a novel Voronoi-tessellation-based method were manufactured. A different approach [115] provided the benchmarking of SLM and robocasting, as manufacturing methods of scaffolds from commercially pure titanium (CP-Ti). The values of compressive yield strength 75 MPa and effective elastic modulus in compression 7 GPa were shown by the SLM-made scaffold, the values closer to those of the cortical bone as compared to robocasting, whereas the robocasted scaffold presented higher ALP activity than SLM-made scaffolds. The SLM has several advantages and shortages as compared to the electron beam melting (EBM). As shown in [27], the SLM processes produced peak temperatures of about 2000–2500 K, and high cooling rates of about 10^4^ K/s in the fabrication of Ti-6Al-4V were applied. EBM generates a similar peak temperature range, but the high build temperature of 600–750 °C decreases the cooling rate locally. The thermal behavior during SLM processes resulted in an acicular α′ martensite microstructure and high tensile stresses, whereas the high build temperature involved in the EBM process led to an α + β lamellar microstructure free from residual stresses. Despite a huge number of attempts, the questions of whether the SLM can produce scaffolds at high geometric accuracy of struts in each part of the scaffold and how the size of the scaffold affects this accuracy still remain. Such a problem is crucial for securing the free flow of body fluids, transport of nutrients and oxygen into the scaffold interior, and bone ingrowths.

#### 4.2.2. Selective Laser Sintering (SLS)

SLS, known as one of the powder bed fusion fabrication methods in which powder materials are heated to fusion instead of completely melted, results in a net-shaped implant with high ductility and porosity [116,117]. For example, Liu et al. successfully produced composite titanium-silica scaffolds with complex geometry, and significant human cells (MG63) proliferation was seen over 7 days [118]. In the comparative studies, [119] the authors presented the mechanical properties of sintered Ti-6Al-4V alloy with 75% porosity, closer to the cancellous bone compared to SLM-made scaffolds characterized by the same value of porosity. Some researchers [118] presented the limitation of the SLS process based on process variables, as the laser energies lower than 12 W and higher than 28 W were not suitable for sintering the titanium powder. However, they showed that the SLS method at a laser power of 15 KW, 16 kHz frequency, and scanning velocity of 100 mm/s within 3 h and postheating in 900 °C for 120 min led to an increase of cell culture optical density from 0.1 to 2.4 after 7 days. In a different approach, the authors presented the influence of SLS process parameters on structural behavior, where the size of the nanostructure increased while the scanning speed decreased and the power laser increased [120].

#### 4.2.3. Electron Beam Melting (EBM)

EBM, as another powder bed fusion fabrication method, also gives similar opportunities as the SLM and it can [121] process patient-specific complex designs, obtained either from the computer tomography (CT) scan of the defect site or through a CAD program. For example, in [122], Ti-6Al-4V prostheses with 3D hierarchical (macro/micro-nano) porosity were constructed by electron beam melting. In [123], the triple- and double-layered mesh Ti64-based alloy scaffolds were fabricated. In other research, the Ti-6Al-4V gyroid scaffolds with porosities in the range of 82–85% and three different unit cells of size 2 to 3 µm [124] demonstrated the elastic modulus and yield strength ranged from 637 to 1084 MPa and from 13.1 to 19.2 MPa, respectively. The as-built scaffolds exhibited excellent ductility up to 50% and no sign of fracture up to 20–30% strain under compression [125]. A different study [126] showed the limitation of the EBM method connected with manufacturing precious porous architecture with struts sized below 500 μm in Ti-6Al-4V scaffolds. A similar situation was observed in [74], where SLM-made structures with pore sizes <750 μm were unachievable. It could be caused by high laser power resulting in higher powder sputtering during production. In another approach, Zhao et al. compared the corrosion resistance of Ti-6Al-4V scaffolds manufactured by EBM and SLM method and showed that the corrosion rates of all types of specimens were well below those recommended by standards of the American Association of Corrosion Engineers and that the scaffolds could be applied in vivo [127]. In the last study [128], anisotropic properties with a higher reduced modulus (up to 10%) and nanohardness (up to 30%) in the transverse direction than those in the building direction were exhibited. The surface treatment was seldom applied for EBM produced scaffolds. The Ti-6Al-4V discs prepared by additive manufacturing (EBM) were coated with layers of pectins, calcium-binding polysaccharides derived from citrus and apple, which also contained alkaline phosphatase (ALP), the enzyme responsible for mineralization of bone tissue. ALP-loaded pectin coatings promoted adhesion and proliferation of human bone mesenchymal stem cells (hBMSC) [129].

#### 4.2.4. Laser Engineered Net Shaping (LENS)

LENS belong to the group of direct laser deposition techniques, where the powder is fed through argon pressurized nozzles [130]. Some authors investigated the role of LENS processing parameters on the microstructure, mechanical, and biological properties [131,132]. Others presented the fatigue behavior and failure mechanisms of the LENS process [133], and the influence of changing process parameters in LENS technology on porosity evolution [134]. The obtained structures contained porosity between 17 and 58 vol %, pore size with maximum value of 800 μm, the mechanical strength of 24–463 MPa and a low Young’s modulus of 2.6–44 GPa [131] or the modulus of porous Ti components ranged between 2 and 20 GPa for the open porosity between 55% and 27%, respectively [132]. Furthermore, Young’s modulus and 0.2% proof strength of the porous Ti samples having 35–42 vol % porosity was found to be similar to those of human cortical bone [132]. Additionally, all studies indicated good cell adhesion, differentiation, and proliferation of LENS-printed scaffolds.

#### 4.2.5. Fused Deposition Modeling (FDM)

In [135], a simple extrusion-based 3D printing FDM technique was developed to produce porous Ti6Al4V scaffolds under ambient environmental conditions. 3D printed Ti-6Al-4V scaffold with a pore size of ~500 μm and total porosity of ~58% was achieved. The scaffold exhibited ~13% shrinkage after sintering, resulting in a strut diameter of ~348 μm with persistent interparticle voids. The compressive strength and elastic modulus values were 39.58 MPa and 450 MPa, comparable to cancellous bone mechanical properties. In vitro cytocompatibility assessment of a scaffold using mesenchymal stem cells revealed extensive cellular coverage on scaffold surface and differentiation toward bone cell lineage. In vivo studies by scaffold implantation in rabbit femur for four weeks and eight weeks exhibited the scaffold’s ability to promote osseointegration and tissue integration through bone ingrowth as evidenced by micro-CT.

#### 4.2.6. Direct Ink Writing (DIW)

DIW, which is also known as robocasting belongs to the material extrusion method, where the colloidal inks are directly extruded from a nozzle [136]. The Ti-6A-l4V alloy mixed with maltodextrin powder was printed with an inkjet printing technology at 1400 °C sintering temperature as the homogenous and gradient scaffolds of porosity over 20%. Effective elastic moduli together with uniaxial compression strength for homogeneously and gradient porous designs were found to be 2.2 GPa and 3.0 GPa, and 57% and 45%, respectively, and as of 47 and 90 MPa of compression strength, values being intermediate between those for cancellous and cortical bones [137]. In similar research [138], the 3D printed Ti-6Al-4V scaffolds were made. After the printing process and drying, the components were sintered at 1400 °C. Highly porous titanium scaffolds (with porosity up to 65 vol.%) were produced and different geometries were printed. The equiaxed grain structure of the produced scaffolds allowed for compression yield strength higher than similar structures produced by energy deposition AM technologies. The compression yield stress ranged from 110 to 130 MPa depending on the geometry of the scaffold. Finally, in [139], a new thermoset biopolymer was proposed which would act as a binder for DIW of titanium artificial bone scaffolds to manufacture porous titanium scaffolds with evenly distributed and highly interconnected pores ideal for orthopedic applications. The scaffolds exhibited an effective Young’s modulus similar to that of human cortical bone and possessed superior strength.

#### 4.2.7. Metal Injection Molding (MIM)

The MIM is another method to produce the titanium scaffolds [140] when combined with space holder techniques. The possibility to produce the scaffolds with porosity over 10% up to 60%, with low elastic modulus 4–22 GP and well-interconnected pores, made of Ti and Ti-6Al-4V alloy, was reported. Using the same technique [141], particles of HAp were blended with a titanium powder and used to produce Ti foams in combination with a space holder. Incorporation of high levels of HAp into the Ti foams induced brittleness in the structure and reduced the load-bearing ability of the titanium foams, but adding it in small amounts, to 2%, was found to increase the yield strength of the Ti foams from 31.6 MPa to 50.9 MPa.

#### 4.2.8. 3D Fiber Deposition (3DF)

3DF deposition is based on forcing Ti slurry through the syringe nozzle using a 3D bioplotter machine. The slurry is plotted on a stage as a fiber, which rapidly solidifies by drying, and the scaffold is fabricated by layering a pattern of fibers. After deposition, the obtained Ti-6Al-4V scaffolds were dried for 24 h at RT and sintered under a high vacuum at 1200 °C for 2 h. By varying spacing and fiber laydown patterns, different Ti alloy scaffolds of the low, middle, and high porosity, double-layered, and with gradient porosity were produced [142].

The comparison of the different additive manufacturing technologies available for the fabrication of titanium scaffolds is shown in Table 3.

### 4.3. Effects of Manufacturing Errors on Properties of Ti Scaffolds

All AM techniques, which use significant heat amounts, can produce serious discrepancies between perfect design and real geometry [27]. Such danger is particularly significant for scaffolds possessing high porosity ad thin inner walls. However, so far, investigations have been rather focused on process optimization to obtain parts perfectly matching the CAD models than on an assessment of the influence of manufacturing errors on properties of scaffolds. Despite that, based on present knowledge, the effects of such discrepancies on mechanical, biological, and chemical properties may be considered. The number, magnitude, and volume of imperfections are not excessive, and for SLM and proper design and manufacturing, the geometrical error may be less than 3% [143]. On the other hand, for the scaffolds additively manufactured with the triple periodic minimal surface (TPMS), the porosity was 12% lower and compressive moduli 15–24% different than those of designed values because of imperfect bonding and partially melted powders [144].

The main imperfections can be divided into exterior and interior ones [27,145]. The external errors are geometrical incorrectness of designed scaffold (change of dimensions, and density) and warping (buckling, change of shape). The source of this manufacturing error is the appearance of high tensile stresses. To avoid this, the manufacturing process is optimized and the upper limits of distortions are set up. Otherwise, the scaffolds cannot be applied. The proper geometry and fatigue resistance are often retained by annealing post-treatment. To prevent buckling, the supports are used during SLM manufacturing. The precise and optimal topology design and optimization are prevalent to diminish the imperfections [21]

Another imperfection is an excessive roughness of the surface. Sometimes, at smooth parts (dental foundations), the roughness is not allowed and must be removed by polishing. In other cases, when using titanium scaffolds as bone implants, rough surfaces enhance the fixation between an implant and bone. Loose powder on the surface is undesirable and should be removed, e.g., by chemical etching [146].

The imperfections may result from both material and process determinants [19,25]. The chemical composition, melting temperature, flowability, heat conduction ability, density, and morphology of the applied powder influence the roughness, but also the geometrical accuracy. The processing parameters such as the laser power and spot diameter, nozzle speed, substrate feed rate, and temperature affect the metal liquid temperature and convection, cooling rates, and temperature gradients. The laser power affects the quality of melt tracks, and the random partial fracture of the melt tracks might appear if the laser power is too low. The scanning interval determines the size of regularly arranged pores. With the increase of the thickness of sliced layers, the density of scaffolds became lower, the contour of the melt tracks became apparent, and the structure became loose [146]. The scan tracks on the top surface morphology changed from clear and uniform mode to disordered mode with the increase of scan speed. A higher scan speed led to the “balls” phenomenon [147]. On the side surface morphology, the melt flow and the overlap of molten pool and balls could be observed. The dimensional accuracy may demonstrate itself as the size shrinkage in the building direction and the periphery spreading effect in the horizontal direction.

The optimization of the initial process parameters may minimize the number of inherent defects, and further thermomechanical treatment may decrease residual stresses, adjust the microstructure [148], and result in a disappearance of sometimes observed martensite [149,150].

In the case of Ti alloys, the processing parameters decide whether equiaxed or elongated columnar grains or both, may appear, even if they might possess a perfect microstructure. However, the grains formed during the AM process are smaller near the interface with the substrate as compared to the subsurface layers. The mechanical performance of the scaffolds is determined rather by the microporosity than microstructure [115]. The lack-of-fusion pores and cracks are the most often observed defects [17]. The pores (voids) at the interface between two subsequent layers are called interlayer porosity or lack-of-fusion porosity and are caused by insufficient energy input. The pores can be generated by the gas bubbles trapped from an environment; this is known as intralayer porosity. Pores are sites at which the cracks may initiate under residual stresses arisen at excessive heating and cooling rates experienced during the AM process by coalescence of pores [151].

The mechanical properties are influenced by the microstructure of the titanium scaffold and its imperfections. The manufacturing errors may decrease strength, ductility, hardness, toughness, fatigue limit, and wear resistance [25]. The optimization of the process and post-treatment is difficult. Using a small beam diameter with high laser power can cause material evaporation and keyholes due to concentrated energy and overheating at a small spot. Therefore, increasing the laser spot size allows using high power lasers without overheating, but this may compromise the precision and surface roughness of the parts [28]. The strength of additively manufactured Ti parts is also dependent on the build orientation [17,152,153]. In [71], the static compressive properties and fatigue lives of the octahedron scaffolds were superior to those of tetrahedron ones.

The desired biological properties need a proper design of a titanium scaffold that requires an appropriate pore shape, pore size, and porosity. All these variables can affect biological performance, such as cell adhesion and proliferation, nutrient transportation, and bone ingrowth. The grain size also seems important [24]. The struts (cell units) are necessary for the flow of cells and nutrients, but they may be also considered as imperfections of a structure. The shape of the unit cell is important for mechanical behavior. The gyroid scaffolds have a higher compressive and tensile strength than the BCC scaffolds. On the other side, the permeability of the gyroid scaffolds, deciding on biological transport, is much lower than that of the BCC ones [154]. As concerns the biological behavior, for both types of unit cells, octahedron, and tetrahedron, cells spread better and displayed more filopodia on scaffolds with greater pore size, but cell proliferation was superior in the octahedral unit cell [71]. Therefore, increasing porosity may enhance the biological processes, but it can decrease the stiffness and strength drastically. This problem is solved by a variety of topology optimization techniques [88]. Despite that, if the manufacturing errors do not drastically decrease the pore size and change the cell unit geometry, their effects on biological behavior seem negligible.

The effect of geometric mismatch and inherent defects on the corrosion behavior of Ti materials is relatively moderate and depends on the AM technique. The potentiodynamic polarization tests showed that the corrosion resistance of the SLM specimen was the best under the low electric potential and that of the EBM specimen was the best under the high electric potential. The crevice corrosion resistance of the EBM specimen was the best, and the corrosion resistance of the SLM specimen was the lowest in the immersion test. On the other side, both scaffolds, fabricated with either EBM or SLM, had good corrosion resistance and were suitable for implantation in vivo [127]. On the contrary, the iron scaffold demonstrated much lower corrosion resistance than the wrought material [155]. Nevertheless, the surface protection of titanium scaffolds is proposed by anodization [156] or by phosphate coatings [157].

## 5. Titanium and Its Alloys for Manufacturing of Scaffolds

In general, Ti and titanium-based alloys show better biocompatibility and mechanical properties compared to stainless steel. Among titanium alloys, the Ti-6Al-4V alloy has been widely used as an orthopedic biomaterial due to its good corrosion behavior and mechanical properties. However, to lower elastic modulus and minimal contents of toxic elements such as aluminum and vanadium, new biocompatible β-Ti alloys with β stabilizing alloying elements (Mo, Si, Ta, Sn, Zr) were recently developed. According to the literature, β-type Ti alloys demonstrate better mechanical properties, due to the moduli closer to those of human bone in comparison to α-type Ti alloys and α + β-type Ti alloys, as well as better biocompatibility due to the non-toxic nature of β-stabilizers [158]. For example, the elastic modulus of human cortical bone is about 30 GPa, while those of Ti–6Al–7Nb and Ti–6Al–4V are about 110 GPa and 112 GPa, and that of Ti-24Nb-4Zr-8Sn is below 50 GPa. To obtain desired mechanical and biological properties, the selection of the appropriate alloying elements to add to β-type Ti alloys is required. However, it is also worth noticing that Mo, Zr, Ta, and Nb elements have a higher density than Ti, and possess high melting points leading to the deterioration of the alloy properties. What is more, Mo, Zr, Ta, and Nb are also expensive. As to decrease the cost of β-Ti alloy, low-cost alloying elements, such as Fe, Mn, Sn, and Cr are used [159]. In Table 4, the mechanical, structural, and biocompatible properties of titanium alloys used for AM scaffolds are presented.

Most researchers have obtained a highly porous structure with a reasonably high compressive strength that mimics the morphology of the replaced bone. According to the literature, commercially pure titanium (CP-Ti) and Ti-6Al-4V alloy are the most used titanium materials in biomedical implants [161]. Some authors presented the advantages of CP-Ti scaffolds. In particular, CP-Ti is characterized by a lower elastic modulus compared to Ti-6Al-4V alloy. The low elastic modulus improves the biomechanical compatibility by reducing the stress shielding effect. In [166], the uniform porous CP-Ti structure was obtained while using freeze-casting, with porosity from 71 to 52 vol %, pore size from 362 to 95 μm, and the compressive strength and stiffness from 57 to 183 MPa and from 1.3 to 5.0 GPa, respectively. With an increase of Ti content from 15 to 25 vol %. [107], the polymeric sponge replication method made it possible to obtain a CP Ti scaffold with macropores ~365 μm, the compressive strength of 84 MPa, and a porosity equal to 66%. While in [167], using the same fabrication method, a similar porosity of about 70 vol % was obtained, but a much lower compressive strength of 18 MPa. Following that, in further research, micro-arc oxidation was proposed as post-treatment. Other researchers used titanium-tantalum (Ti-Ta) alloys as promising materials for such applications due to their high strength-to-density ratio. However, the great differences in density (4.5 and 16.6 g/cm^3^, respectively) and melting point (1670 and 3020 °C, respectively) between Ti and Ta could lead to strong inhomogeneity during the alloy formation [168]. Nevertheless, some previous work has shown that SLM is capable of producing Ti-Ta porous scaffolds. For example, in [169], the effects of tantalum (Ta) on microstructure, mechanical properties, and corrosion behavior of SLM-printed Ti-Ta scaffold were investigated. The increasing Ta addition promoted the formation of the β phase and led to the increased value of tensile strength from 641 to 1186 MPa and the microhardness from 257 to 353 HV. In another study, Huang et al. [162], when fabricating scaffolds of titanium-tantalum (Ti-Ta) alloys with 0, 10, 30, and 50 wt.% of tantalum by SLM method, observed the biological response similar to that of Ti-6Al-4V and commercially pure titanium. Ti-30Ta was characterized by the lowest modulus. Furthermore, some reports indicated that the addition of Nb to Ti promoted apatite formation and improved the proliferation of MG63 cells compared to titanium without Nb addition [163]. For example, Liang et al. [170] obtained satisfactory bioactivity results, with a higher level of ALP expression in each treatment group of Ti-Nb alloys with varying Nb contents (0–45 at.%), compared to the pure Ti. In a different approach, Fangxia et.al. [171] used SLS with postheating to obtain Ti-Mo open porous microstructure. According to this report, the addition of the Mo element had a positive effect on body pH balance, revealed a strong β-stabilizing effect, and enhanced corrosion resistance. Ti-Mo SLS-made scaffolds showed porosity ranged from 36 to 61 wt.pct., an elastic modulus from 3.28 to 8.51 GPa, and compressive strength from 243 to 370 MPa. Several studies on AM scaffolds were performed using the SLM method and Ti-6Al-4V (ELI) alloy in the last period. ELI grade is known to have excellent biocompatibility. In [9], the SLM-made alloy with triply periodic minimal surfaces of 74% porosity and ∼900–1000 μm pore diameter were characterized by high strength (169 MPa) and low stiffness (5.09 GPa). In another research, Wang et al. [172] showed that for porous Ti-6Al-4V scaffold with functionally graded architecture, the cell proliferation rate from day 4 to day 7 was 140%, whereas for the uniform structures, it was only 90%. In [173], the bioactivity of SLM-made Ti-6Al-4V scaffolds was improved by forming TiO_2_ nanotubes on the surface through two-step anodization and loading mesoporous bioactive glass into TiO_2_ nanotubes. Another research group [174] successfully obtained an SLM-made integrated trilayered scaffold with titanium (Ti-6Al-4V)-Mesh-Cage, filled with the autogenous cancellous bone for the bone graft to the osteochondral defect. The results showed that the original defect was fully covered by cartilagelike tissue only 3 months after the in vivo test. Vlad et al. [175] used Ti-6Al-4V and SLS with thermal treatment and sandblasting to obtain metallic scaffolds filled with hydroxyapatite bioactive matrix. The researchers proved greater osteogenic performance with the fully mineralized bone after 6 months, compared to titanium scaffolds without ceramic matrix. As some authors reported the adverse effect of the presence of the α-phase in Ti-6Al-4V enhancing brittleness and reducing the fatigue life of the components, the new, β-type Ti-24Nb-4Zr-8Sn alloy with a significantly lower modulus of 42–50 GPa compared with other conventional titanium alloys (100–120 GPa), was achieved by AM technologies. For example, Liu et al. [176,177] presented porous architecture produced by EBM with 70% of porosity, strength 35 MPa, characterized by better mechanical properties and at least twice the strength-to-modulus ratio of Ti-6Al-4V porous components with the same porosity level. The same authors manufactured Ti-24Nb-4Zr-8Sn alloy scaffolds where the strength reaching 51 MPa [178]. Other research groups [179] proposed the fabrication of Ti-10Mo-xFe scaffolds using a powder metallurgy process as the potentially low-cost process to manufacture porous structure. The addition of Fe and Mo to Ti alloys enhanced their mechanical strength and reduced their elastic modulus [158,165]. The studies indicated that Ti-10Mo-5Fe revealed the highest compressive strength (2392 MPa) and strain (43%), and elastic modulus (91 GPa) low as compared to CP-Ti and some other Ti-based alloys. Other authors [89] used Ti alloy with Nb and Zr addition to not only enhance the mechanical properties but also improve the corrosion and wear resistance. Through powder metallurgy, they obtained Ti-35Zr-28Nb scaffolds with a much lower compression yield strength (230.5 MPa) and elastic modulus (6.9 GPa) compared to other results [179], with values closer to those of human bone. The corrosion resistance was higher (corrosion rate ~0.91 mm/year) than the values obtained for CP-Ti (1.77 mm/year) with similar porosity (around 50%). Similarly, other researchers [113] obtained SLM-manufactured Ti-35Zr-28Nb scaffolds with a porosity of 50% with significantly better electrochemical behavior compared to that exhibited by CP-Ti. However, the SLM manufacturing led to lower elastic modulus (1.3 GPa) and compressive yield strength (58 MPa) than the values presented in [179]. In a different research, Luo et al. [164] used Ti-30Nb-5Ta-3Zr alloy, as another low-modulus biomedical β-Ti alloys with nonallergic elements, to manufacture porous structure. The authors proved better biocompatibility, lower stress shielding, and a better bone healing rate of SLM-printed alloy than the comparative Ti-6Al-4V samples.

Table 5 demonstrates the materials applied for scaffolds together with fabrication techniques.

## 6. Structural Factors Influencing the Mechanical Properties

Van Bael et al. [72] presented the dependence between the compressive stiffness and pores’ characteristics (size and geometry). Comparing those values with bones’ properties (trabecular: 0.1–2.942 GPa, cortical: 14.7–34.3 GPa), it could be noticed that the stiffness of both the rectangular (1.3–2.03 GPa) and the triangular geometry (0.4–2.8 GPa) had values within the range for trabecular bone, while the hexagonal design with the 500 µm pore size (11.26 GPa) was approaching the lower range for cortical bone. Considering only scaffolds made by the SLM, in [71], effects of different unit cell types (tetrahedron and octahedron) and pore size (500 μm and 1000 μm) on fatigue properties were studied. It was found that octahedron scaffolds exhibited superior static mechanical properties, longer fatigue lives, and higher fatigue strength in comparison to those of tetrahedron ones. It is noteworthy that the porous structure was often built using diamond lattice pore units [200,201,202]. In [126], the Ti-6Al-4V porous scaffolds of two unit cell geometries (reentrant and cubic) fabricated using EBM showed that samples with the cubic unit cell geometries, with struts oriented at an angle of 45° to the loading direction, and exhibited higher stiffness than samples with the reentrant unit cell geometry at equivalent relative densities. A cubic scaffold with a pore size of 0.6 mm obtained high mechanical properties with compressive strength approaching 70 kN. In [203], the effect of cell geometry was investigated with the diamond, rhombic dodecahedron, and truncated cuboctahedron struts. In another study, Zhang et al. [45] fabricated the diamond lattice pore unites with constant pore size, varied struts, and different porosity 66.1–79.5%. The elastic moduli and compressive strength increased with the greater support struts and were found to vary between the corresponding mechanical properties of cancellous and cortical bone. Due to its high value of compressive strength equal 140 MPa as well as proper elastic modulus 5.15 GPa, the biomimetic implant was made based on a diamond pore structure with a strut dimension of 400 µm for in vivo experiments. Other research groups showed that the continuous functionally graded porous titanium scaffolds could be also manufactured by the SLM based on the Schwartz diamond unit cell and the strut size of 483–905 μm. The elastic modulus and yield strength of the scaffolds could be tailored in the range of 0.28–0.59 GPa and 3.79–17.75 MPa, respectively, by adjusting the graded volume fraction [112].

The triply periodic minimal surfaces (TMPS), especially gyroid structures, are characterized by better compressive fatigue resistance correlated with lower stress concentrations [184]. For as-built by SLM Ti-6Al-4V alloy scaffolds, the relations between porosity (50–90%), compressive and tensile strength, and stiffness were observed. As concerns the mandibular constructs (CMC) made of titanium scaffolds, higher magnitudes of tensile strains were observed for models with 0.2 mm and 0. 4 mm strut diameter, both having 0.5 mm interstrut distance (ID). The maximum principal tensile strains were higher in the CMC models with 0.5 mm ID as compared to those having 0.3 mm ID. Comparatively, the scaffolds with lesser ID (0.3 mm) resulted in higher stiffness, thereby evoking lower principal strains in the CMC models. Moreover, considering the weight of the scaffolds, the CMC models having 0.3 mm ID with 0.2 mm SD and 0.5 mm ID with 0.6 mm SD is the most appropriate for a patient [201]. In [204], with a fixed strut diameter of 0.45 mm and a mean cell size of 2.2 mm, a tetrahedral structural porous scaffold was designed for a simulated anatomical defect derived from the CT data of a human mandible. Geometric and mechanical comparisons between the initial and optimized scaffold showed that the optimized scaffold exhibited a larger porosity, 81.90%, as well as a more homogeneous stress distribution. These results demonstrate that tetrahedral structural titanium scaffolds are feasible structures for repairing mandibular defects and that the proposed optimization scheme can produce superior scaffolds or mandibular reconstruction with better stability, higher porosity, and lower weight. In [205], to reduce the implant stiffness, open-porous designs in two geometrical dimensions were investigated (twisted design). The elastic modulus of the scaffolds varied between 3.4 and 26.3 GPa and scaffolds porosity ranged from 54 to 60%. Results showed a clear influence of the unit cell orientation on elastic modulus, compressive strength, and strain. Different experimental data were obtained by Speirs et al. [74], who investigated three unit cells (diamond, triangular, hexagonal) at three different pore sizes (1000, 750, and 500 μm). Stiffness and compressive strength were shown to increase twice with decreasing pore size for hexagonal and diamond designs, whereas for the triangular design, the compressive stiffness increased as pore size increased. The highest porosity and ultimate compressive strength were obtained for scaffold with triangular pores with a size equal to 750 μm, whereas the highest values of compressive stiffness at a pore size of 1000 μm. The scaffold production with a unit size of 500 μm was unsuccessful because of the occlusion effect. Cheng et al. [51] focused on isostructural foams based on a human trabecular bone template. The varying porosity (15–70%) of allotropic pore shape resulting in compressive modulus ranging from 2.58–3.69 MPa. Fousova et al. [57] chose a rhombic dodecahedron with 200 µm in size and strut diameter 300 µm as an elementary cell of porous parts. It was shown that samples with gradient porosity structures containing a dense core and 2 mm porous surface with a porosity of 61% met the best mechanical properties (E 30.5 GPa) similar to bone values. Wysocki et al. [206] fabricated scaffolds with a bimodal pore size (200 μm core and 500 μm shell). In this study, the compressive strength (447 MPa) and elastic modulus (42.7 GPa) was a few times higher than values for human cortical bone and other reported architectures with the same diamond unit cell and similar dimensions. The bimodal structure was also studied in [207], where the gradient porosity was generated by the multiplication of body-centered cubic unit cell. The results indicated that the optimal structure contained the smaller pores in the core (~900 µm), and bigger pores in the shell (~1100 µm) part of the scaffold due to the improvement of mechanical properties inside and cells adhesion outside of construction. Also in [208] radially graded structures with diamond unit cells and four zones of various porosities (91.3 for inner layer-10.5% for outer layer) were proposed.

In Table 6, the selected published data of mechanical parameters, pore shape, pore size, and porosity are presented.

## 7. Structural Factors Influencing the Biological Properties

### 7.1. Biocompatibility and Bioactivity

Biocompatibility is understood as the capability of a material to perform with an appropriate host response in a specific application [210], and bioactivity is defined as the ability of apatite to form in phosphate-containing environments with osteoconductive and osteoinductive properties [211]. In [212], for the small specimens, 5 mm round, 5 mm in diameter and 4 mm in height, made of titanium, of porosity 50% and pore size over 300 µm, after 6 weeks of in vivo studies, the pores subjected to mineralization resulting in a decrease of porosity to about 6%. No bone cells were observed inside the pores smaller than 10 µm. Another research showed [200] that porous Ti-6Al-4V scaffolds demonstrated apatite-forming ability after an additional surface treatment such as alkali–acid–heat or hydrothermal. Khodaei et al. [213] heated porous titanium scaffolds at 600 °C, finding the hydrophilicity and apatite formation ability of titanium surface at heat treatment up to 240 min. In [214], three-dimensional TiO_2_ scaffolds were functionalized on the surface of the scaffolds using alkaline phosphatase (ALP), which was chosen in this study due to its important role in the bone mineralization process. After 28 days in simulated body fluid (SBF), ALP coated titania scaffolds exhibited increased hydroxyapatite formation. Another surface treatment helpful in an enhancement of biomineralization was the deposition of three-layer bioglass within the titanium scaffolds [215]. In [216], pure zeolite silicate coatings on titanium scaffolds promoted the formation of mineralized nodules. Another deposit resulting in higher apatite forming ability for porous titanium was forsterite/poly-3-hydroxybutyrate nanobiocomposite [81]. SLM manufactured porous Ti-6Al-4V scaffolds with diamond pore structure were post-treated using a combination of alkali–acid–heat (AH) and hydrothermal treatment (HT) to obtain the TiO_2_ layer and HAp coating. The apatite-forming ability test and in vitro cell culture assay resulted in the highest apatite formation and significantly greater adhesion and differentiation of mesenchymal stem cells (MSCs) on the HT/AH-porous Ti6Al4V compared to the scaffolds without HAp-coating [200]. In [217], the pulsed reverse electrodeposition was used to obtain calcium phosphate (CaP) coatings doped with chlorhexidine digluconate on an additively manufactured CP-Ti scaffold. The result showed the surface of scaffolds was covered by plate-like and whisker-like calcium phosphate crystals with a Ca/P ratio of 1.30. Another research [218] group used Ga(No_3_)_3_ as a new therapeutic agent to promote bone formation. The results of the apatite-forming ability in SBF solution showed the continuous and homogeneous apatite layer with a Ca/P ratio of 1.7 after five days of incubation. The longer the incubation time was, the greater thickness of apatite coating appeared, while with a higher concentration of gallium nitrate (100 mM) the apatite layer was formed faster and could be already noticed on the 3rd day.

As can be observed, titanium scaffolds are often subjected to different surface treatments. However, small specimens are applied and no research results on apatite deposition inside the scaffolds have been given what could be of capital importance for an assessment of total mineralization and the efficiency of different methods. The investigations of mineralization are made on the surface, and exceptions are rare.

### 7.2. Osteoconductive and Osteoinductive Properties

#### 7.2.1. In Vitro Studies

There has been a great amount of research in vitro on titanium scaffolds, without or with surface modification. When the selective laser melting was employed to fabricate the trabecularlike porous scaffolds with porosities 49–74%, the surface of the SLM-fabricated Ti-6Al-4V scaffolds was favorable for osteoblasts’ adhesion and migration because of microscale pores and ravines [114]. In [219], the porous titanium implants with over 90% of porosity showed sufficient cell penetration. In [220], the osteogenic and angiogenic responses to macroporous scaffolds coated with silicon substituted HAp (SiHAp) and decorated with vascular endothelial growth factor (VEGF) showed that SiHAp would stimulate the proliferation of MC3T3-E1 pre-osteoblastic cells, whereas the adsorption of VEGF would stimulate the proliferation of EC2 mature endothelial cells. The composite scaffold consisting of porous Ti part filled with chitosan/HAp sponge [221] improved osteoblast adhesion and morphology and increased proliferation and ALP activity. For stem cell engineered bone with calcium-phosphate-coated porous titanium scaffold, significantly increased cell proliferation and ALP activity was found [222]. Brecevich et al. [223] using human bone-marrow-derived mesenchymal stem cells (hMSCs) observed the strongest adhesive affinity and cell viability at porosities between 50% and 70%. The increased levels of BMP2 expression were found for porosities between 50–70%, whereas increased levels of VEGF, osteocalcin, and osteoprotegerin expression were found on scaffolds at porosities between 70–80%. In [224] the HAp/TiO_2_ surface allowed greater adsorption of serum proteins and further enhancement of the ALP activity of MC3T3-E1 osteoblasts. In another research study [225], the electrophoretic deposition of calcium phosphate nanoparticles on the Ti-6Al-4V scaffolds resulted in improving both adhesion and growth of hMSCs, and the osteogenic differentiation behavior of hMSCs. Modification of titanium foam by hydrothermal treatment following an Mg^2+^ or Ca^2+^ ion-substitution process [226] affected the cell morphology, viability, gene, and protein expression of mesenchymal stem cells (MSCs) grown on the surface of nanostructured titanium. In [227], TiO_2_ nanostructures obtained by hydrothermal treatment on three-dimensional porous titanium scaffolds surface facilitated the cell culture medium to penetrate the inner pores of the scaffold. In [228], the behavior of human osteoblastic cells cultured on dense and porous titanium and Ti-35Nb alloy showed no significant difference in several biological properties. In another research study [61], the highest metabolic cell activity and proliferation in the scaffold was obtained at pores of 400 to 620 µm in size, a porosity of 75%, and an open-porous pyramidal unit cell. Similar results were obtained by Taniguchi et al. [70], where the in vivo test for the scaffolds with diamond structure and pore size of 600 μm showed significantly higher fixation ability than those with a pore size of 300 μm and 900 μm. In [229], the preliminary in vitro studies presented good proliferation of osteoblast in scaffolds with pore size equal 350 μm. In [230], Ti scaffolds with meshes of 0.8 mm showed higher osseointegration compared with 1 mm mesh. When investigating the effect of porosity for titanium scaffolds [231], several viable cells for 60% and 73% porous titanium were more numerous than at 87% porous titanium. The last scaffold demonstrated the highest osteocalcin production, and each of the titanium scaffolds showed higher osteocalcin production compared to βTCP (tricalcium phosphate) scaffolds. In [232], the Ti-6Al-4V scaffolds of different porosity and pore size (low, 334.1 μm pore size with 55.4% porosity; middle, 383.2 μm pore size with 65.2% porosity; and high, 401.6 μm pore size with 78.1% porosity) were investigated. The three types of porous Ti-6A-l4V scaffolds were inclined to promote cell proliferation, whereas cell differentiation and bone ingrowth into the porous scaffolds were biased to the porous titanium with relatively large pores and porosity (middle and high). In [233], the authors showed that post treatment like sandblasting or sandblasting/acid etching of scaffolds significantly improved their osseintegration. The trabecularlike porous scaffolds with full irregularity and higher porosity exhibited enhanced cell proliferation and osteoblast differentiation at an earlier time, due to their preferable combination of small and large pores with various shapes [114]. Li et al. [113] showed the cell viability and adhesion of the SaOS2 cells on the SLM-manufactured Ti-35Zr-28Nb scaffolds with various structures. The results indicated the cell viability at no significant difference between FCCZ, FBCCZ, and control, good cell adhesion, and proliferation in both groups (FCCZ, FBCCZ) on a fully covered surface after 14 days. The cell adhesion density was in the following order: control > FBCCZ > FCCZ. In [200], ALP activity of MSCs cultured on the surfaces of SLM manufactured porous Ti6Al4V increased for all the samples, with the highest values obtained by specimens subject to both hydrothermal and alkali–acid–heat post-treatment, compared to the samples treated only by the only alkali–acid–heat or without any post-treatment. Li et al. [186] filled the functionally graded titanium (FG-Ti) with osteoinductive silk fibroin (SF) sponge Ti by freeze-drying and examined the biocompatibility of the obtained structure (FG-Ti+SF) by seeding the top surface of each scaffold with a rat osteoblast cell suspension. The results showed that functionally graded composite scaffolds consisting of FG-Ti filled with SF sponge indicated a higher degree of cell attachment, viability, and proliferation (from 4 to 7 days) when compared to FG-Ti scaffolds. In comparison, Zhao et al. [234] used gel casting based on 3D printing with electrolysis reduction to fabricate porous tantalum scaffolds. The results of cell culture indicated that the Ta scaffold was nontoxic, cell proliferation was the highest after the 4th day. In a comparative study of SLM and robocasting method of manufacturing porous scaffolds, [115] the obtained CP-Ti scaffolds possessed high cytocompatibility with no significant differences between the two types of scaffolds, but with a higher rate of ALP activity observed for the Rob-scaffolds. Similar results were obtained in [138] with fibroblasts well attached and spread on the surface of Ti-6Al-4V scaffolds manufactured by direct ink writing (DIW) technology. To improve surface osteogenic activity, Zhao et al. [173] developed Ti–6Al–4V scaffolds with TiO_2_ nanotube arrays (Ti-NTs) and mesoporous bioactive glass (Ti-NTs-MBG). After the 7th day of in vitro assay, the cells cultured on the 3D-Ti-NTs-MBG scaffolds presented no significantly different proliferation rates, when compared with those on the 3D-Ti-NTs scaffolds. In [217], cell behavior on CP-Ti scaffold coated by calcium phosphate (CaP) showed that the addition of CHX decreased cell adhesion in comparison to the scaffolds with CaP coatings.

#### 7.2.2. In Vivo Studies

A novel biomimetic porous titanium implant with good osseointegration was prepared by freeze-casting and thermal oxidation [235] as measured by cell proliferation assay, ALP activity assay, X-ray examination, and hard bone tissue biopsy. In [236], the submicron-thin HAp-coated titanium fiber mesh scaffolds showed, after 21 weeks, expression of osteocalcin, and in vivo bone formation. The newly formed bone in HAp-coated scaffolds mostly restored bone continuity, strong integration of the bone and HAp-coated scaffolds. When comparing Ta and Ti [237], both scaffolds were in favor of hBMMSCs proliferation and osteogenic differentiation. Porous scaffolds implanted in the femur bone defects rabbits in vivo showed that both porous scaffolds were beneficial to the bone ingrowth and bone implant fixation. As rough or nanostructured surfaces, the graded porous structures designed using triply periodic minimal surface models to mimic the biomechanical properties of bone promoted early osteogenesis and osteointegration on bone formation in vivo [238], for the pores ranged from 100 µm to about 700 µm and porosity about 50%. Tests in vivo on rats showed [239], for porous titanium with the average 3D pore sizes of the three groups as of 188 µm, 313 µm, and 390 µm, and at a porosity of 70% that the differentiation stage of cells on the porous titanium with the most narrow pores as compared with the smooth solid titanium plate was more inclined to promote cell differentiation at the initial stage, whereas cell proliferation and bone ingrowth were biased to titanium with larger pores. In a research work [240], porous titanium scaffolds coated with diamondlike carbon (DLC) were produced, and their ability to form biocomposites was evaluated through in vivo experiments. At 24 weeks after surgery, the bone tissue grew through the whole scaffold depth, and the bone composition (Ca/P ratio) in the peripheral pores was close to the composition of the compact bone. The important role of macro-, micro-, and nanoroughness on biological behavior was investigated for Ti scaffolds [190]. On the macroscale, surface roughness can contribute to a dramatic increase in the bone/implant contact area, which maximizes bone ingrowth. Microscopically, again in titanium, surface roughness promoted the production of osteoprotegerin (OPG), TGF-b1, VEGF-A, FGF-2, and angiopoietin-1. The scaffold supported the adhesion and growth of human fetal osteoblast cells. The crystalline structure seems to be a minor factor [190]. In a different approach, a barium titanate (BaTiO_3_), with or without low-intensity pulsed ultrasound (LIPUS) as a mechanical wave promoting bone regeneration, was used to modify the surface of a porous Ti-6Al-4V scaffold. In vitro results of bone marrow mesenchymal stem cells, namely adhesion, proliferation, and gene expression were significantly higher for BaTiO_3_/Ti, Ti + LIPUS, and BaTiO_3_/Ti + LIPUS groups compared to the Ti. The ALP activity was also higher in the BaTiO_3_/Ti + LIPUS group than in the BaTiO_3_/Ti and Ti + LIPUS groups. The results were in line with the in vivo experiments of implantation in large segmental bone defects in the radius of rabbits, where osteogenesis and osseointegration at 6 and 12 weeks after implantation were significantly better for these implants compared to porous Ti condition [193]. Other research groups [175] studied the SLS manufactured Ti-6Al-4V scaffolds filled with hydroxyapatite bioactive matrix. The in vitro results proved good adhesion, proliferation, and viability of human cells on the composite scaffold. Additionally, the in vivo results confirmed the better osteogenic behavior of the studied samples, compared to those without bioactive matrix. In another study [241], the authors took advantage of improving the surface bioactivity by coating titanium scaffolds with autologous platelet-rich plasma (Ti-PRP) prepared by the traditional method (TrdPRP) and freeze-dried method (FDrPRP). It was shown that in all cases autologous platelet-rich plasma had a positive effect on scaffolds biocompatibility, and osteogenic differentiation ability. Moreover, compared with the TrdPRP, the FDrPRP exhibited better properties in terms of their ability to improve cell activity and osteogenic differentiation, as well as the increase of bone regeneration.

### 7.3. Antibacterial Effects

Antibacterial scaffolds have not been often proposed. In [242], a porous titanium scaffold was treated by the direct oxidation method. Strontium-containing gelatin microspheres were synthesized and deposited on the surface to postpone strontium release and to initially release gentamicin. In [243], the layers composed of nanosilver particles (AgNPs), CaP nanoparticles, and combinations of both were formed on metallic scaffolds. The AgNPs at a concentration of approximately 0.02 mg/cm^2^ hindered bacterial growth. Finally, in [244], silver loaded gelatin microspheres were incorporated into porous titanium. The high antibacterial ability against both *Escherichia coli* (*E. coli*) and *Staphylococcus aureus* (*S. aureus*), was demonstrated. In [122] Ti-6Al-4V prostheses with 3D hierarchical (macro/micro-/nano)porosity were constructed by electron beam melting followed by micro-arc oxidization and silver nanoparticles (AgNPs) immobilized. In another study, Ti scaffolds were produced by DMP and coated with chitosan gel (Ch), with various concentrations of AgNO_3_ (Ch+Ag) via the EPD method, or biofunctionalized with vancomycin (Ch+Vanco). The implants demonstrated antibacterial behavior of 99.9%, while 100% was observed in Ch+Vanco implants. However, in vivo implantation in the rat tibia indicated that all implants were colonized with bacteria after 28 days, among them the Ch+Vanco group significantly reduced bone infection as compared to other implants (Ch-only, Ch+Ag) [245]. In [217] multifunctional CaP coating loaded with chlorhexidine digluconate (CHX) at 0.75, 1.5, and 3 mM was successfully applied on DIW-manufactured CP-Ti scaffolds to reduce bacterial adhesion. The biological tests showed that the as-coated scaffolds reduced bacteria adhesion by 73% for *Staphylococcus aureus* and 70% for *Escherichia coli*. Results showed that CHX was more effective against Gram-positive bacteria than Gram-negative ones, thus the minimal required concentration of CHX should be 1.5 mM for inhibition of both bacteria growth. Additionally, the authors determined a significantly higher total amount of CHX loaded to the coating (136.8 μg/mL) compared to the previous research presented by that research group (CaP coating, with a total amount of 45 µg/mL CHX loaded in the coating) [246]. Moreover, 52% of CHX released during the first 12 h and noticeable release in more than 1 week afterward was noticed (previous research showed more than 80% of the CHX release in less than 6 days). Rodriguez et al. [218] successfully manufactured CP-Ti scaffolds by the DIW method. To obtain the antibacterial properties, the researchers developed gallium-doped Ca titanate coatings via the traditional thermochemical treatment (TT). The analysis of the cytotoxicity of the treated Ti surface showed antibacterial effect only against Gram-negative strains *Pseudomonas aeruginosa* and *E. coli*, and only in the first hours. Both were characterized by a quick release of Ga ion and after 48 h no bacteria growth inhibition was noticed. Additionally, the minimal concentration of Ga required for inhibition effect against *E.coli* bacteria was 100 mM.

In Table 7, the selected published data of biological in vitro and/or in vivo properties of scaffolds are presented.

## 8. Structural Factors Influencing the Chemical Properties

The most studied chemical property of titanium scaffolds is their corrosion resistance in a biological environment. Generally, the corrosion resistance is high, but at inflammations states, the pH value drastically decreases, and the corrosion rate increases. In scaffold, the important problem is the presence of long and narrow holes, which may provoke the localized corrosion [247]. In [248], porous titanium coatings were studied in a dynamic physiological environment. Then, a titanium-based implant was fabricated by plasma spraying. The studies of potentiodynamic polarization and electrochemical impedance spectroscopy show that the pores in the porous titanium negatively affect corrosion resistance and the flowing electrolyte can increase the corrosion rate of all titanium samples. Therefore, special coatings have been proposed. Liu et al. [249], combining alkali treatment and natural cross-linker, procyanidin, created submicron-porous structure and immobilized type I collagen on the surface of Ti-24Nb-4Zr-8Sn alloy scaffold with interconnected porosity. The hybrid layer, an outer submicron-porous layer, and an inner dense layer were formed. The proposed surface treatment enhanced corrosion resistance. In [110], for the 75% porous titanium, the sol–gel coating of pore walls with hydroxyapatite conferred a decrease of corrosion current from 670 to 39 µA for porous titanium structures in a 0.9% NaCl solution at 37 °C. The problem of electrochemical behavior in a body was discussed in [10]. Despite the fact that the titanium alloy is characterized by excellent corrosion resistance, the porous structures are more vulnerable to body fluid. The electrolyte isolated into porous structures, together with limited oxygen supply, contribute to a lower ability to passively form layers. However, according to [10], the interconnectivity of porous structure minimize this problem. Samples with porosity equal 15% and 24% showed corrosion behavior, while the porosity of 33% did not. Wei et al. [250] indicated the increase of electrochemical activity in phosphate-buffered saline (PBS) of Ti-10Mo alloy samples with the increasing porosity. In their study, porous Ti-10Mo alloy possessed higher corrosion resistance in PBS compared to porous CP-Ti with similar porosity. For SLM-manufactured Ti-35Zr-28Nb scaffolds with various structures (FCCZ and FBCCZ), the corrosion results indicated low corrosion rates [113].

## 9. Conclusions

This review briefly summarizes recent progress in additive manufacturing techniques of porous Ti and its alloys dedicated for biomedical applications, focusing especially on structural and material determinants influencing the mechanical and biological properties. First, a brief introduction to the biological background comprising the formation and regeneration process of bone is presented. Afterward, the main requirements for bone scaffolds are discussed. Next, fabrication methods of the titanium scaffolds focus mostly on additive manufacturing techniques with their advantages and disadvantages based on the latest research are comparatively studied. Afterward, titanium materials and their alloys are described. Finally, the scaffold’s structural factors influencing the mechanical, biological, and chemical properties are discussed.

Porous titanium structures with different pore shapes and biomedical properties that are similar to those of the human bones, minimizing the stress shielding and improving the longevity of implants, can be successfully fabricated by additive manufacturing techniques. Many aspects affect the mechanical and biological responses of porous structures, such as the manufacturing methods and their process parameters, design (pores shape and size, porosity), material selection, post-treatment. Highly porous scaffolds are found to have lower mechanical properties, higher permeability, and better cell ingrowth, especially when the larger pores are situated on outer surfaces. The increasing strut diameter leads to a higher load-bearing capacity. The obtuse angles of struts improve mechanical properties, while a larger number of them improve cells’ bridging effects. A compromise between bone ingrowth, vascularization, mechanical strength, and permeability, shows that the optimum pore size is supposed to range between 300 and 600 μm. Titanium and its alloys are widely used for manufacturing porous scaffolds. However, the latest research is mostly focused on investigating porous structures with new β-type titanium alloys (containing Mo, Si, Ta, Sn, Zr elements), which are characterized by better mechanical properties and lower amounts of toxic elements. Among manufacturing methods, SLM-made scaffolds present superior properties compared to those produced by conventional methods, especially casting, with only a minor reduction in maximum deformation strain. The SLS process is characterized by the high temperature of the process, while the EBM, due to the vacuum condition of the process, leads to the lower number of defects of manufactured parts, compared to SLM-made structures. Besides many advantages of 3D printing, the microstructural defects in the builds are still a challenge. Thus, several surface modification methods are used to improve mechanical and biological properties. Heat and acid-treatment, anodizing, coatings contained CaP, HA, chitosan, or antibiotics are commonly used to enhance surface roughness, corrosion, and wear resistance, as well as improve the osseointegration process.

Despite indisputable advantages of AM as a technology for titanium scaffold fabrication, some considerable challenges and disadvantages in this area exist. Future research should consider the following direction: Research standardization, Hybrid 3D printing, a combination of 3D and 2D printing.

The mechanical and biological properties of scaffolds cited in the literature are quite different even for similar materials. The large variations in experimental procedures may be correlated with different AM process conditions, different sizes of titanium powder particles used in the fabrication process as well as the difference in research methodology. The completed database contains categorization of existing results, supported by medical examination should be evaluated.

Although titanium alloys can meet the mechanical requirements, their bioactivity should be improved. The surface treatment which usually enhances the bioactivity of titanium materials, in the case of scaffolds is a big challenge due to the difficulties correlated with homogenous incorporation of bioactive elements into the porous structure. Thus, a hybrid printing, contains 3D printing (enhanced mechanical stability) and parallel 3D printing allows the incorporation of biochemical molecules (cells, grown factors) directly into a 3D printed scaffold should be evaluated.

Another approach to minimize the disadvantages of 3D printing is a combination of 2D and 3D printing methods. Similarly to the issues mentioned above. The surface treatment is difficult inside the scaffolds, especially the removal of loose powders after AM leads to a decreasing of the interconnectivity of scaffolds, thus using 3D printing combined with 2D nanostructuring of each of the layers during the formation of the 3D structure should be evaluated.

Finally, due to the complexity of factors influencing the behavior of scaffolds, the computer-enhanced design with patient-specific finite element models of bones should be evaluated.

## Figures and Tables

**Table 1:** Mechanical properties, porosity, and density of human cortical and trabecular bone.

Bone	Trabecular	Cortical	References
Porosity (%)	50–90	3–12	[46,47,48,49]
Density (g/cm^3^)	0.30 ± 0.1	1.85 ± 0.06	[47]
Stiffness (GPa)	0.1–2.942	14.7–34.3	[4,50]
Tensile strength (MPa)	10–20	50–150	[34,47]
Compressive strength (MPa)	4–12	130–193	[47,51]
Young’s modulus (GPa)	0.02–0.5	3–30	[46,47]

**Table 2:** Structural, mechanical, and material requirements of a bone scaffold.

Parameters	Specification	References
Structural requirements		
Pore shape	Cubic, rhombic dodecahedron, truncated cuboctahedron, rhombicuboctahedron, diamond, truncated cube	[60,73]
Optimal pore size	300–600 µm	[39,51,67]
Porosity	50–90%	[46,67]
Mechanical requirements		
Compression strength	10–200 MPa	[34,78]
Young’s modulus	0.02–30 MPa	[47,78]
Material requirements		
3D architecture Biocompatibility Biodegradability Radiolucent Easily shaped or molded Nonallergic and noncarcinogenic Strong enough to endure trauma Stable over time Osteoconductive		[5,79]

**Table 3:** Comparison of the different additive manufacturing technologies available for the fabrication of titanium scaffolds [2,13,88,117,142].

Methods	Advantages	Disadvantages	Resolution (μm)	Costs	Post-Treatment
SLM	-high-precision complex parts -no support structure required -mechanical properties better than SLS (due to the level of heating during printing)	-worse resolution than SLA, SLS, EBM -unmelted powders may be trapped inside parts -high temperature of the process	250–700	$$	may be necessary due to the presence of partially sintered metal on the struts
SLS	-processing speed is high -good mechanical properties, but worse than for SLM and EBM -high utilization of powder materials, -no support structures required, -superior dimensional accuracy, -efficient resource consumption	-high molding principles, high molding conditions, and high cost, -limited part size, particle sizes -the powders are not fully melted	76–100 μm	$$$	may be necessary due to the presence of partially sintered metal on the struts
EBM	-superior mechanical properties due to the complete melting of the powders -higher resolution than SLM	-the high temperature of the processed areas, -unmelted powders may be trapped inside parts	50–100 μm	$$$$	may be necessary due to the presence of partially sintered metal on the struts
FDM	-low cost -increased thermal conductivity of parts, -reduced deformation during fabrication	-anisotropic and poor mechanical properties compared with the SLM, EBM -high temperature of the printing has a negative effect on bioactive additives. -possible manufacturing problem with excessively acute angles	250–370	$	poor surface quality, required additional treatment
LENS	-excellent mechanical properties -better efficiency, cooling effect, and parts refabricating capability compared to SLM, EBM, SLS -possible integration of different materials, -effective time of the process -highly controlled microstructure	less complex models in comparison with SLM, EBM, SLS	250	$$$	poor surface quality, required additional treatment
MIM	-high printing speed -high manufacturing precision compare to SLS -suited to mass production -low cost -low melting temperature	-low dimensional stability and reproducibility	-	$	Required additional treatment
DIW	-low temperature of the process -flexible manufacturing -high storage modulus and excellent shape retention capacity, -good level of resolution -low cost	requires support structures when manufacturing complex architectures	<200 μm	$	May be required additional treatment
3DF	-preparation time is reduced -each layer may have a different fiber diameter, thickness, fiber space, and fiber orientation -parametric analyses are possible	-low resolution	-	$$	High surface quality

“$”—costs of AM technology, where $ means the cheapest, and $$$$ the most expensive technology.

**Table 4:** The mechanical, structural, and biocompatible properties of titanium alloys used for AM scaffolds.

Material	Modulus (GPa)	Tensile Strength (MPa)	Alloy Type	Mechanical Properties	Biocompatibility	References
CP-Ti	100–120	240–550	α			[160,161]
Ti-Ta	200	-	α	Modulus much higher compare to cortical bone The increasing of Ta addition increases tensile strength and decrease modulus	+, Elements such titanium, niobium, tantalum after implantation in rats exhibited good biocompatibility	[160,161,162]
Ti-35Nb	80	382	β	Nb element reduces the elastic modulus	+, The addition of Nb to Ti promoted apatite-formation	[160,161,163]
Ti-7.5Mo	80	665	α + β	Better corrosion resistance compared to CP-Ti, Ti-6Al-4V	+	[160]
Ti-6Al-4V	112	895–930	α + β	Modulus much higher compared to cortical bone	+, contains toxic elements V, Al	
Ti-13Nb-13Zr	79–84	973–1037	Metastable β	Nb and Zr addition enhance mechanical properties, corrosion, and wear resistance, Nb elements reduce the elastic modulus	++, better compared to α and α + β alloys, the addition of Nb to Ti promoted apatite-formation	[160,161]
Ti-24Nb-4Zr-8Sn	42	-	β			[160,161]
Ti–10Nb-3Mo	-	-	β	Nb and Mo enhance mechanical properties, Nb element reduces the elastic modulus		[160,161]
Ti-20Nb-15Zr	-	-	β	Nb and Zr addition enhance mechanical properties, corrosion, and wear resistance, Nb element reduces the elastic modulus		[159]
Ti-35Zr-28Nb	-	-	β			[159]
Ti-30Nb-5Ta-3Zr	90	700	β			[164]
Ti-10Mo-xFe	91	-	α + β	addition of Fe and Mo to Ti alloys enhanced their mechanical strength and reduced elastic modulus		[158,165]

“+”: level of biocompatibility.

**Table 5:** Materials applied for scaffolds together with fabrication techniques.

Material	Manufacturing Method	References
CP-Ti	SLM/robocasting	[115]
	SLM	[71,128,162,180]
	Freeze-casting	[102,103,166,181]
	Sponge replication process	[107,109,110,167]
	Powder metallurgy	[80,81,83,84,85,93,96,97,98,100,101,106]
	Injection molding	[140,141]
	Direct ink writing	[139]
	LENS	[131]
Ti-xTa	SLM	[162,169]
Ti-xNb	SLM	[170]
Ti-xMo	SLS	[171,182]
Ti-6Al-4V	SLM	[9,112,172,173,174,183,184,185,186,187,188,189]
	SLS	[119,175,190]
	EBM	[27,122,125,126,127,191,192,193,194]
	LENS	[133,134,195]
	Direct ink writing	[137,138]
	3DF deposition	[142]
	(Dynamic) freeze-casting	[105,196,197]
	FDM (customized)	[135]
	Injection molding	[140]
Ti-13Nb-13Zr	SLM	[74]
Ti-24Nb-4Zr-8Sn (Ti2448)	EBM	[176,177]
	SLM	[178]
Ti–10Mo-xFe	Powder metallurgy	[179]
Ti–10Nb-3Mo	Powder metallurgy	[99]
Ti-20Nb-15Zr	Sponge replication process	[108]
Ti-35Zr-28Nb	Powder metallurgy	[198,199]
	SLM	[113]
Ti-30Nb-5Ta-3Zr	SLM	[164]

**Table 6:** Mechanical properties of porous Ti and its alloys.

Material	Pore Shape	Pore Size ^1^ (µm)	Strut Size ^1^ (µm)	Porosity ^2^ (%)	Mechanical Properties ^3^			References
					Young’s Modulus (GPa)	Compressive Stiffness (MPa)	Ultimate Compressive Strength (MPa)	
Fully dense								
Ti-6Al-4V (SLM)	-	-	-	0.8	118.9	1040	1842	[57]
Ti-6Al-4V (hot-rolled)	-	-	-	0	117.2	879	1835	
Fully porous								
	Triangular	500	200	31.63	N/A	2840	N/A	[72]
		1000	200	19.17	N/A	453	N/A	
Ti6-Al-4V	Hexagonal	500	200	57.66	N/A	11,256	N/A	
		1000	200	29.75	N/A	3881	N/A	
	Rectangular	500	200	33.35	N/A	2038	N/A	
		1000	200	16.95	N/A	1300	N/A	
		650	200	79.5	1.22	N/A	36.45	[45]
		650	250	76.3	2.00	N/A	56.63	
Ti-6Al-4V	Diamond	650	300	72.6	3.02	N/A	85.81	
		650	350	67.9	3.79	N/A	109.20	
		650	400	66.1	5.15	N/a	140.26	
Ti-6Al-4V	Rhombic dodecahedron	200	300	79.2	N/A	19.0	21.5	[57]
	Triangular	1000	200	34.88	N/A	5426	102.87	[74]
		750	200	52.32	N/A	3418	198.81	
	Hexagonal	1000	200	33.03	N/A	1623	55.38	
Ti-13Nb-13Zr		750	200	34.86	N/A	3256	112.59	
	Diamond	1000	200	25.81	N/A	868	21.12	
		750	200	34.98	N/A	1912	59.87	
		177	628	15.0	3.69	N/A	N/A	[51]
Ti-6Al-4V	-	383	454	37.9	3.52	N/A	N/A	
		653	305	70.0	2.58	N/A	N/A	
	Primitive	679	260	65	6.4	295.4	N/A	[154]
Ti-6Al-4V	Gyroid	574	220	65	7.6	392.1	N/A	
	Body-centered cubic	882	600	65	4.7	216.0	N/A	
	Face centered cubic	2000	300	87.3 (83.2)	1.1	N/A	27	[113]
Ti-35Zr-28Nb	Face and body-centered cubic	2000	300	78.9 (49.9)	1.3	N/A	58	
CP-Ti	Cubic			54.9	7.22		75.04	[115]
Functionally graded structure –bimodal pore size								
CP-Ti	Diamond	Core 200 Shell 500	Core 100 Shell 200	56–67	42.7	N/A	447	[209]
Porous shell + Dense core								
Ti-6Al-4V	Rhombic dodecahedron	200 (porous shell: 1 mm)	300	37.9	65.1	578	1072	[57]
		200 (porous shell: 2 mm)	300	62.1	30.05	257	393	
Dense shell + Porous core								
Ti-6Al-4V	Rhombic dodecahedron	200 (porous core:	300	48.4	47.6	422	579	[57]

^1^ strut and pore size: in the model; ^2^ porosity: open, ^3^ type of loading: compression; compression strength in the axial direction, N/A: not available.

**Table 7:** The selected published data of biological properties of scaffolds.

Material and Manufacturing Methods	Surface Treatment	Apatite Forming Ability	Antibacterial	In-Vitro Assay		In-Vivo Assay		References
				Cells	Results	Model	Results	
Ti-6Al-4V, SLM	AH HT/AH	The highest for HT/AH treatment	-	MSCs	The best adhesion and differentiation after HT/AH treatment	-	-	[200]
CP-Ti, Space holder technique	Heat treatment for various time	Increasing with the rising heat treatment time up to 240 min	-	-	-	-	-	[213]
TiO_2_, foam replica method	ALP using self-polymerization of dopamine	An increased HAp formation for ALP- coated titania	-	-	-	-	-	[214]
Ti-6Al-4V, hydrothermal synthesis	Zeolite silicalite-1 coatings by secondary growth method	Formation of mineralized nodules noticed	-	Rabbit bone marrow mesenchymal stem cells (r-BMSCs)	Significantly enhanced the attachment and proliferation of r-BMSCs	-	-	[216]
Ti-6Al-4V, SLM	-	-	-	MG63 cells	Enhanced osteoblasts’ proliferation and differentiation for trabecular-like scaffolds with the full irregularity (0.5) and higher porosity (63 or 74%)	-	-	[114]
CP-Ti, freeze-casting	HF/HNO_3_ acid treatment with various time condition	-	-	Preosteoblast cell line (MC3T3-E1)	high number of cells attached to the pore surface after 12 min of treatment	-	-	[219]
Ti-6A-4V, EBM	Ti/Ti+SiHAp+VEGF obtained by dip-coating method	-	-	Murine preosteoblastic MC3T3-E1/mature endothelial cells	VEGF stimulated the proliferation of endothelial cells on the surface. The stimulated proliferation of preosteoblasts on SiHAp coated scaffolds	Osteoporotic sheep model	SiHAp+ VEGF: a significant increase in ossification and angiogenesis degree	[220]
CP-Ti, EBM	Chitosan/HAp sponge by freeze-drying	-	-	Rat osteoblasts	Improved osteoblast adhesion, proliferation and alkaline phosphatase (ALP) activity	-	-	[221]
CP-Ti, SLM	Various structures	-	-	Marrow-derived mesenchymal stem cells (hMSCs)	The strongest cell adhesion for porosities 50–70%, at lower porosities the increased levels of DNA and ALP	x	x	[223]
CP-Ti, Sintering	HAp/TiO_2_ subject to AA treatment	-	-	MC3T3-E1 osteoblasts	HAp/TiO_2_ improved adsorption of serum proteins and enhanced the ALP activity	-	-	[224]
Ti-6Al-4V, EBM	Calcium phosphate nanoparticles (CaPNPs) by electrophoretic deposition	-	-	hMSCs	Improved cell attachment, proliferation, and differentiation, increase of ALP activity	-	-	[225]
CP-Ti, freeze-casting	Thermal oxidation	-	-	MG63 osteosarcoma cells	With increasing coculture time from 1 to 5 days, cell proliferation increased with co-culture time from 1 to 5 days. Significant increase in cell proliferation and differentiation after thermal treatment.	Rabbits	No loosening or bone resorption, and bone ingrowth and osteogenesis were found for modified and unmodified scaffolds. Thermal modification improved the differentiation of osteoblasts in the pores.	[235]
CP-Ti, SLM	TiO_2_ obtained by HT method	-	-	BMSCs	Enhanced cell adhesion and spreading on the nanowire-functionalized scaffold.	-	-	[227]
Ti6Al4V, SLM	Gradient porous structures	-	-	-	-	Mini pigs	Stimulated bone ingrowth achieving a stable interface after 5 weeks after implantation (the push-out force 1100 N–1300 N).	[238]
CP-Ti, SLM	Various pore sizes: 300, 600, 900 μm	-	-	-	-	Rabbits (fixation ability for the cortical bone of the rabbit tibia/bone ingrowth for cancellous bone in the rabbit femur)	At 600 μm, a significantly higher fixation ability in 2 weeks than the other implants. After 4 weeks, sufficiently high fixation ability for all porosities.	[70]
CP-Ti, SLM	nano-SiHAp 0.8 and 1 mm cell size	-	-	-	-	Femur bone defects of White Californian male rabbits	Better osseointegration of nano SiHAp coated specimens higher osseointegration at 0.8 mm cell size	[230]
Ti-6Al-4V, EBM	Various pore sizes (low, middle, and high) and porosities	-	-	MC3T3-E1	No differences were observed in cell adhesion and morphological characteristic. ALP activity significantly higher after 7 and 14 days for middle and high pore size	Rabbits with distal femoral defects	New bone formation higher for middle and high pore size after 12 weeks after implantation	[232]
Ti-35Zr-28Nb, SLM	FCCZ and FBCCZ structures	-	-	Human osteoblastlike cells (SaOS2)	No significant difference in cell adhesion, proliferation, and viability. Good cell adhesion after 14 days. Cell adhesion density in order: control > FBCCZ > FCCZ.	-	-	[113]
Ti-6Al-4V, SLM	Varying irregularities (0.05–0.5) and porosities (48.83–74.28%)	-	-	MG63	Cells number higher in specimens with smaller irregularities and lower porosities. Good cytocompatibility in all groups Higher cell density at lower porosities and for higher irregularities Higher ALP activity for high irregularities and high porosities	-	-	[114]
CP Ti, direct metal printing	Chitosan gel/chitosan gel+Ag/chitosan gel+ vancomycin	-	Ch + 50 mM Ag and Ch + 100 mM Ag reduced the number of *S. aureus* both at 24 h and 7th day in 99.9%. Ch + vancomycin completely killed bacteria.	MG-63	Ch + Ag coatings reduced the number of attached MG-63 cells after 24 h	Rat tibia	Ch + vancomycin coatings reduced the infection rate more as compared to chitosan-only coatings. Ch + Ag coatings did not indicate the antibacterial effects.	[245]
Ti-6Al-4V, SLM	Silk fibroin	-	-	Rat osteoblast	Cell attachment, growth, and proliferation on the FG-Ti scaffold improved by adding ECM-like SF sponge in the porous scaffold	-	-	[186]
Ta, Gel casting	-	-	-	L929	Uniformly attached to the scaffolds and the significant cell proliferation observed after 4 days	-	-	[234]
Ti-6A-l4V, DIW	Sintering	-	-	Human fibroblast	Fibroblasts well attached and spread on the surface. The best results after the 14 days	-	-	[138]
Ti-6Al-4V, EBM	BaTiO_3_ deposition LIPUS treatment	-	-	MSCs Rabbit primary BMSCs	Cells adhesion, proliferation, and gene expression significantly higher after surface treatment.	Rabbits	Osteogenesis and osseointegration in 6 and 12 weeks improved after implantation for surface-treated scaffolds.	[193]
CP Ti, SLM/robocasting	-	-	-	SAOS-2 osteogenic cell line	The high cytocompatibility of SLM-made, and Rob-scaffolds. Higher ALP activity in Rob-scaffolds.	-	-	[115]
Ti-6Al-4V SLM	Ti-NTs and Ti-NTs-MBG	-	-	hBMSCs	Improved adhesion and proliferation rate of Ti-NTs and Ti-NTs-MBG compared to Ti scaffolds. No significant difference in biological activity between Ti-NTs and Ti-NTs-MBG.	-	-	[173]
Ti-6Al-4V, SLS	HAp bioactive matrix	-	-	Human osteoblasts	Cell adhesion, proliferation, and viability are not negatively affected with time by compositional factors. Ability to promote and sustain osteogenic differentiation, matrix maturation, and mineralization in vitro.	Transverse and spinous processes of sheep’s	Vertebrae hybrid scaffolds had greater infiltration, with the fully mineralized bone after 6 months than those without bioactive matrix.	[175]
Ti-6Al-4V, EBM	PRP-coated porous Ti	-	-	BMSCs	Significant promotion of BMSCs attachment, proliferation, migration, and osteogenic differentiation	Osteoporosis models	Enhanced bone regeneration and osseointegration	[241]
CP Ti, DIW	CaP coating loaded with CHX	Surface covered by platelike and whisker-like CaP crystal (mainly octacalcium phosphate and brushite)	Reduced bacteria adhesion (73% for *S. aureus* and 70% for *E. coli*). 52% of CHX released during the first 12 h	Sarcoma osteogenic cells (SaOS2)	Adhesion and spreading of cells on coated surfaces. CaP + 1.5 m MCHX considered optimal for reaching a compromise between cell adhesion and antibacterial response	-	-	[217]
CP Ti, DIW	Gallium deposited by thermochemical treatment	Ga improved the nucleation of an apatite layer Ca/P = 1.7 after 5 days	Ga improved an antibacterial effect against Gram-negative bacteria during the first hours, correlated with high initial release of Ga ions	SaOS-2 osteoblast-like cells	Ga improved cells adhesion, proliferation, differentiation, and mineralization.	-	-	[218]

## Abbreviations

3DF	3D fiber deposition
AH	alkali–acid–heat treatment
ALP	alkaline phosphatase
AM	additive manufacturing
CAD	computer-assisted design
CaP	calcium phosphate
CaPNP	calcium phosphate nanoparticles
Ch	chitosan
CHX	chlorhexidine digluconate
CMC	complete mandibular construct
CP Ti	commercially pure titanium
CT	computer tomography
DED	direct energy deposition
DIW	direct ink writing
DLC	diamond-like carbon (layer)
DMLS	direct metal laser sintering
EBM	electron beam melting
FBCCZ	face- and body-centered cubic unit cell with longitudinal struts)
FCCZ	face-centered cubic unit cell with longitudinal struts
FDM	fused deposition modeling
FG-Ti	functionally graded titanium (FG-Ti)
HAp	hydroxyapatite
hBMSC	human bone mesenchymal stem cells
hMSCs	marrow-derived mesenchymal stem cells
HT	hydrothermal treatment
ID	interstrut distance
LENS	laser engineered net shaping
LIPUS	low-intensity pulsed ultrasound
MBG	mesoporous bioactive glass
MIM	metal injection molding
MSCs	mesenchymal stem cells
OPG	osteoprotegerin
PBS	phosphate-buffered saline
PRP	platelet-rich plasma
r-BMSCs	rabbit bone marrow mesenchymal stem cells
SaOS2	sarcoma osteogenic cells
SBF	simulated body fluid
SF	silk fibroin
SiHAp	silicon substituted hydroxyapatite
SLM	selective laser melting
SLS	selective laser sintering
Ti-NTs	TiO_2_ nanotube arrays
TMPS	triply periodic minimal surfaces
Vanco	vancomycin
VEGF	vascular endothelial growth factor
βTCP	beta tricalcium phosphate
